# Adaptive attention-based human machine interface system for teleoperation of industrial vehicle

**DOI:** 10.1038/s41598-021-96682-0

**Published:** 2021-08-26

**Authors:** Jouh Yeong Chew, Mitsuru Kawamoto, Takashi Okuma, Eiichi Yoshida, Norihiko Kato

**Affiliations:** National Institute of Advanced Industrial Science and Technology, AIST Tsukuba Central 1, 1-1-1 Umezono, Tsukuba, Ibaraki 305-8560 Japan

**Keywords:** Mechanical engineering, Human behaviour

## Abstract

This study proposes a Human Machine Interface (HMI) system with adaptive visual stimuli to facilitate teleoperation of industrial vehicles such as forklifts. The proposed system estimates the context/work state during teleoperation and presents the optimal visual stimuli on the display of HMI. Such adaptability is supported by behavioral models which are developed from behavioral data of conventional/manned forklift operation. The proposed system consists of two models, i.e., gaze attention and work state transition models which are defined by gaze fixations and operation pattern of operators, respectively. In short, the proposed system estimates and shows the optimal visual stimuli on the display of HMI based on temporal operation pattern. The usability of teleoperation system is evaluated by comparing the perceived workload elicited by different types of HMI. The results suggest the adaptive attention-based HMI system outperforms the non-adaptive HMI, where the perceived workload is consistently lower as responded by different categories of forklift operators.

## Introduction

Demand for teleoperation systems is increasing due to the emergence of pandemic which essentially changed the social behavior and work pattern in daily life. Physical interactions and contacts between humans are discouraged and digitalization of interactions towards remote or online interactions is accelerated. Teleoperation system is therefore, getting more attention and interest, where the applications range from telepresence systems^1^ in the convenience stores, to teleoperation systems at workplaces, such as teleoperation of heavy machineries at construction sites^2–4^ or industrial vehicles at warehouses^5,6^. However, transition from physical or manned operation to teleoperation is not easy because of issues such as implementation cost, safety, and usability of new teleoperation systems. This usability is typically dependent on the visual stimuli shown on the displays of Human Machine Interface (HMI). In case of teleoperation HMI for heavy machineries such as cranes^2–4^, the recommended visual stimuli usually cover a relatively small working area around the machine itself. Thus, views from an overhead camera covering this working area are consistently recognized as the optimal visual stimuli to facilitate teleoperation of cranes. However, these visual stimuli may not be suitable for different applications which may have different operation characteristics. For example, some applications require multiple tasks such as driving and handling of load. Thus, the attention of operators may need to have multiple perspectives.

To develop an intuitive HMI with good usability for varying applications, one promising approach is to present suitable visual stimuli to operators during operation. For this purpose, prior knowledge of operators’ attention for these applications is necessary, but it is not easy to achieve and existing studies^2–6^ provide no indication on how to identify operator’s attention. For example^3^, proposed teleoperation HMI based on the attention-awareness model which consists of three types of views, i.e. “Focused”, “Ambient”, and “Alerting” views. However, the methods to identify visual stimuli for these views were not explained. Thus, there is still a barrier to apply this model to different applications.

Alternatively, a more common or straightforward approach to develop a teleoperation HMI is to present as much visual stimuli as possible. This method provides high awareness for better operation safety, where multiple fixed visual stimuli which have large coverage of client’s surrounding environment, are presented on multiple displays of HMI^5^. However, operators can be confused and may face difficulties to find the desired visual stimulus, especially if there are multiple machines in case of single-operator-multi-robot operation. It is also possible to develop a teleoperation system using telepresence^6^, where head motion of the operator is tracked, and the optimal view is presented accordingly through the HMD, Essentially, operators can see the environment as if they are present physically. However, this method requires the environment model and sensors to track the states of client and host.

We can therefore conclude that the ideal approach is to minimize the number of visual stimuli to be presented on the display of HMI while ensuring good operation safety. This study proposes a method to identify attention of operators during manned operation of an industrial vehicle, taking the example of forklift. These attentions are then used to select and present optimal visual stimuli on teleoperation HMI. Consequently, it is possible to minimize visual stimuli presented during teleoperation while ensuring operation safety because the presented visual stimuli are expected to be optimal at that instant of work state. As described above, this study intends to answer the following research question, i.e. “When to show what visual stimuli to the operator during teleoperation?”. The first contribution of this paper is the extension of the attention-awareness model^3,7^ to define the three types of views as functions of gaze behavior. With these definitions, it is possible to develop intuitive teleoperation HMI for different applications based on the same model or approach. The second contribution is the extension of the work state transition model^8^, where given the operation input, the corresponding gaze attention are also estimated in addition to the work state.

## Objectives and assumptions

This study focuses on developing an intuitive teleoperation HMI based on human behavior observations. Specifically, the study uses the forklift operation as a case study because the problem is challenging yet remains mostly unexplored for teleoperation. Although fully autonomous forklift systems exist, human intervention is often necessary to supervise and intervene in the event of accidents or difficult situations. More importantly, an intuitive teleoperation system is necessary for semi-autonomous operation like single-operator-multi-robot systems, where operation efficiency can be increased by allowing an operator to supervise multiple machines.

### Assumptions

This section explains two assumptions which are the basis of the development of Adaptive Visual Stimuli (AVS) for HMI of forklift teleoperation. The adaptability of the proposed system is supported by behavioral models which are developed using data from manned forklift operation. Assumption 1 and 2 refers to operation pattern and gaze behavior, respectively.

Assumption 1: A forklift operation typically consists of a sequence of basic work states, where each state is triggered by unique operation pattern which can be discriminated through analysis of operation input vector, defined as a set of input values from the operator.

Assumption 2: At each work state, operators tend to exhibit unique gaze pattern where gaze attention focuses on different area of workspace with varying distribution and transition.

### Objectives

**Figure 1:**
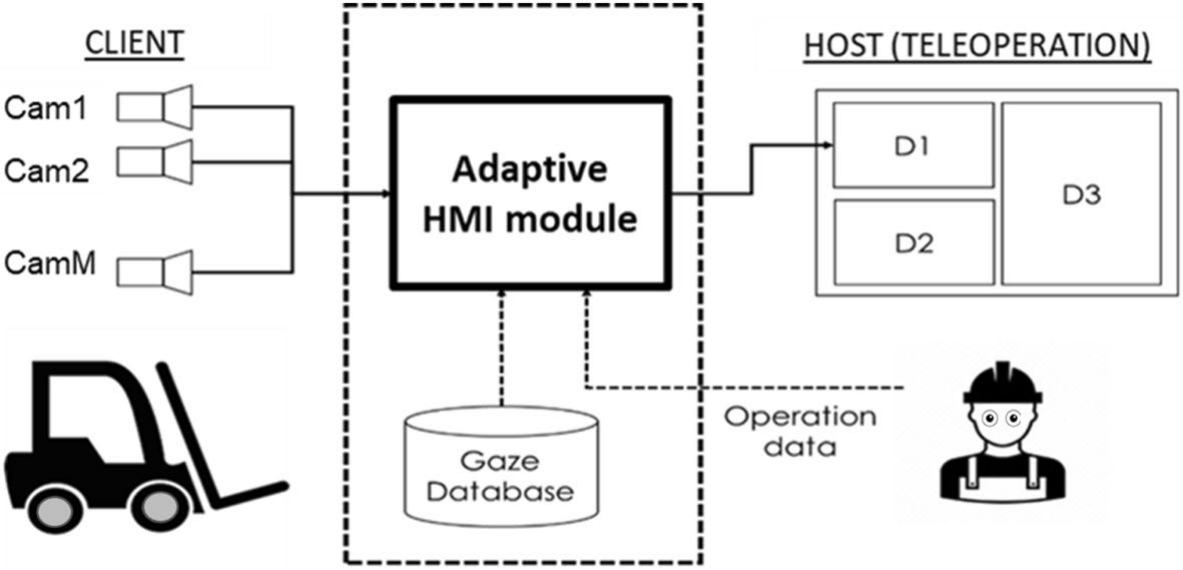
Adaptive attention-based HMI for teleoperation of forklift.

Referring to Fig. 1, D_i_ is defined as the ith image frame which presents one of the views acquired from the cameras mounted on the forklift. Hereafter, D_i_ is referred to as HMI element. Based on the two assumptions in the preceding section, this study aims at developing a novel adaptive attention-based HMI for teleoperation of forklift as illustrated in Fig. 1. The Adaptive HMI module selects the optimal visual stimuli for HMI elements D_1_ and D_2_, where D_1_ = *h*_1_(**u**, **v**, **C**) ∈ {c_1_, c_2_, … , c_M_}, and D_2_ = *h*_2_(**u**, **v**, **C**) ∈ {c_1_, c_2_, … , c_M_}. The operation input vector and gaze attention matrix are represented by **u** and **v**, respectively. The intrinsic and extrinsic camera parameters such as the focal length, position, and orientation, of the cameras mounted on the forklift are represented by **C**. Essentially, functions *h*_*1*_ and *h*_*2*_ select the optimal visual stimulus for D_1_ and D_2_ from a set of views acquired from M cameras mounted on the forklift, where c_i_ is the view of the i^th^ camera.

As described above, the following objectives are defined in this study.

- To develop behavioral models of manned forklift operation using operation pattern and gaze behavior.
- To develop an adaptive HMI for forklift teleoperation.
- To evaluate usability of adaptive HMI from the perspective of perceived workload.

### Acquisition of behavioral data

This section explains the experiment which was carried out to acquire behavioral data of manned forklift operation. The experiment was carried out according to the rules and regulations of National Institute of Advanced Industrial Science and Technology (AIST) of Japan. Informed consents were obtained from all human subject participants and the experiment protocol was reviewed and approved by the Human Factor experiment committee of AIST. All the subjects have forklift operating licenses, and the experiment was participated by 57 subjects from four categories as explained below. The recruitment plan is 15 subjects/category, but the actual number differs due to recruitment difficulty. However, this does not affect behavioral analysis for 3 subject categories.

- 16 Novice with forklift work experience of < 2 years
- 23 Intermediate with forklift work experience of ≥ 2 year and < 10 years
- 17 Expert with forklift work experience of ≥ 10 years
- 1 Instructor with experience as the instructor of forklift training course

**Figure 2:**
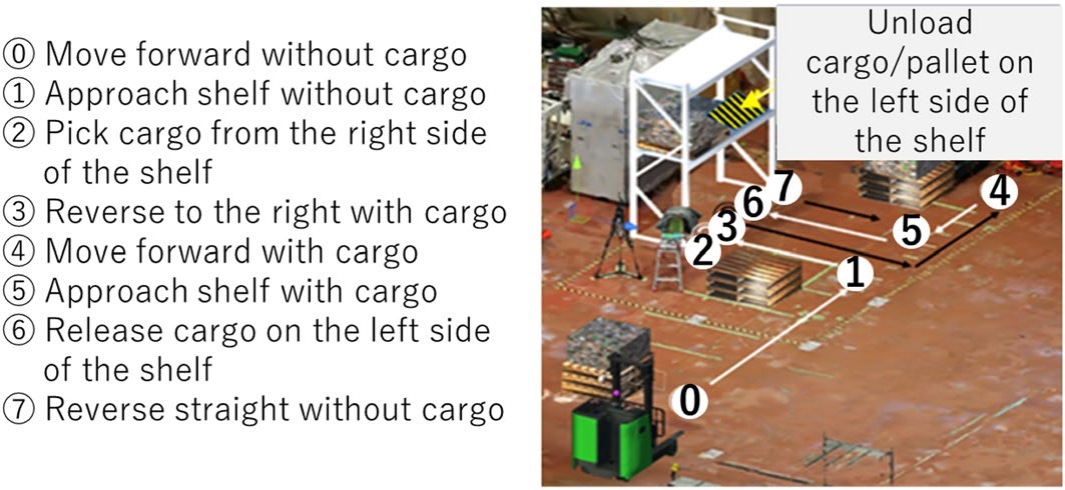
The experiment task consists of basic forklift operations in a virtual environment such as moving forward, backward, approach shelf, loading and unloading.

Each subject performed the experiment task in a virtual environment (see Fig. 2) three times after one training. The task consists of basic forklift operations which are typical in the actual forklift work. The experiment was carried out in the virtual environment using the forklift simulator which was developed in the preceding study^6^. We found that subjects exhibited similar operating behavior compared to performing the same task in the real environment. Based on the results of^6^, the same assumption is made in this study.

Assumption 3: The forklift simulator emulates operation behavior when performing the same task in the real environment.

## Methodology

**Figure 3:**
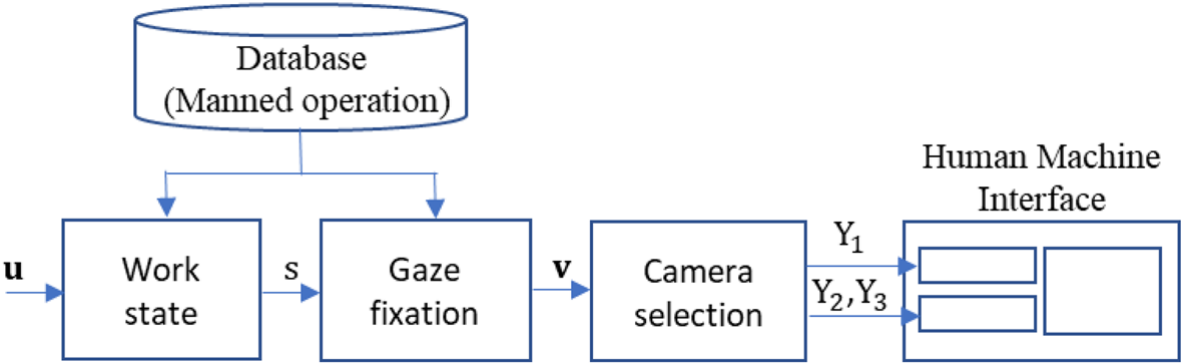
Configuration of the adaptive attention-based teleoperation HMI.

This section explains the configuration of AVS for teleoperation HMI (see Fig. 3), which consists of work state, gaze fixation and camera selection modules. The inputs are operation input vector **u** and the outputs are optimal visual stimuli Y_i_ for i number of adaptive HMI elements D_i_. First, the configuration of HMI elements is elaborated. Then, the development of the work state and gaze fixation models using database of manned forklift operation are explained. Next, the method to select the optimal visual stimuli Y_i_ using gaze attention **v** and camera parameters are explained. Lastly, test conditions of the usability test are elaborated.

### Configuration of HMI elements

**Figure 4:**
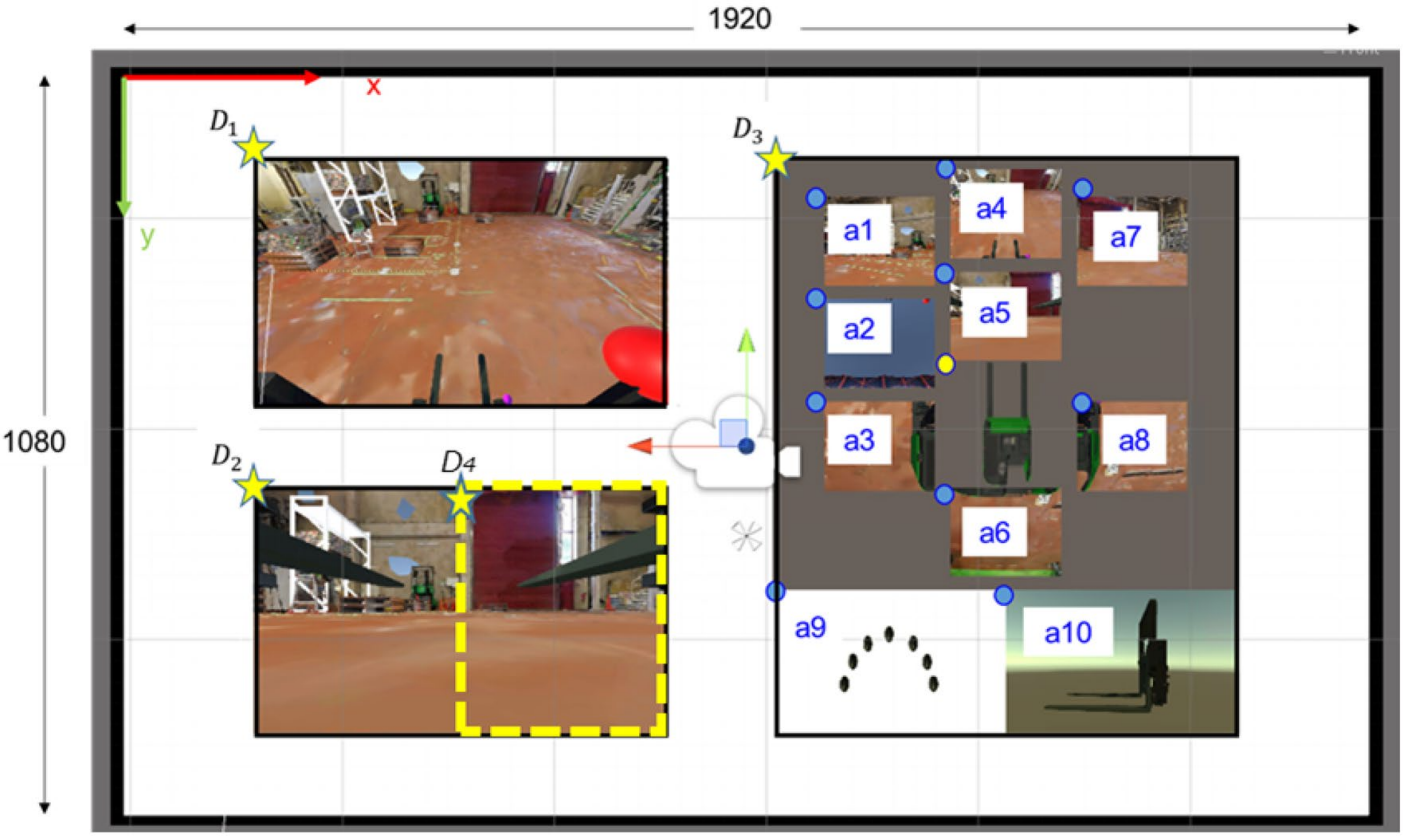
The HMI consists of multiple elements D_1_ to D_4_ implemented on a 27-inch display with 1920 × 1080 resolution (see Appendix B for the details of cameras mounted on the forklift which provide the views for UI elements a1 to a8).

**Figure 5:**
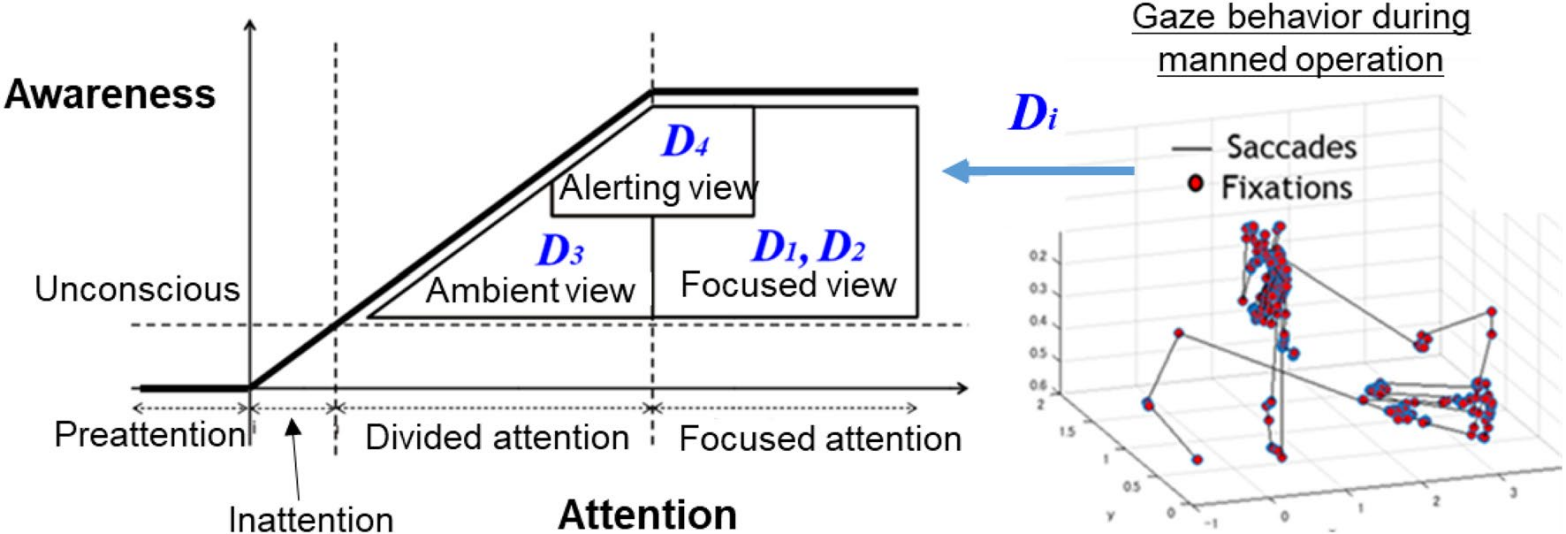
The HMI elements are developed according to the attention-awareness model^3,7^, but the method is extended by defining adaptive views emprically using gaze fixations of manned operation.

The HMI consists of several elements as shown in Fig. 4. The basis of development is the attention-awareness model^3,7^, where three types of views, i.e. “Focused”, “Ambient”, and “Alerting” views, are shown on the HMI. Compared to the preceding studies^3,7^ which defined these views intuitively, this study extended the method by defining each type of view using gaze attention as illustrated in Fig. 5. Specifically, gaze fixations of manned forklift operation are used to select the optimal views for adaptive elements D_i_. This novel approach facilitates implementation of the attention-awareness model to different applications, where definition of these views can be determined empirically. This study mainly evaluates the usability of adaptive views defined by gaze attention, and the layout of these elements are not evaluated.

Referring to Fig. 5, each type of view is represented by HMI elements in Fig. 4 (see Appendix A for details such as sizes and positions of each HMI element).

- Ambient view is represented by non-adaptive HMI element D_3_
  D_3_ shows the views from all cameras mounted on the forklift for operation awareness and safety (see Appendix B for camera positions and orientations for a_1_ to a_8_). Supplementary information such as tire direction and tilt status of the fork are also presented by a_9_ and a_10_, respectively.
- Focused view is represented by adaptive HMI elements D_1_, D_2_
  D_1_, D_2_ are expected to present optimal visual stimuli to the operators so that teleoperation can be performed without having to “search” the HMI. In principle, operators are expected to focus mainly on D_1_, D_2_ if the proposed system is easy-to-use.
- Alerting view is represented by adaptive HMI element D_4_

**Figure 6:**
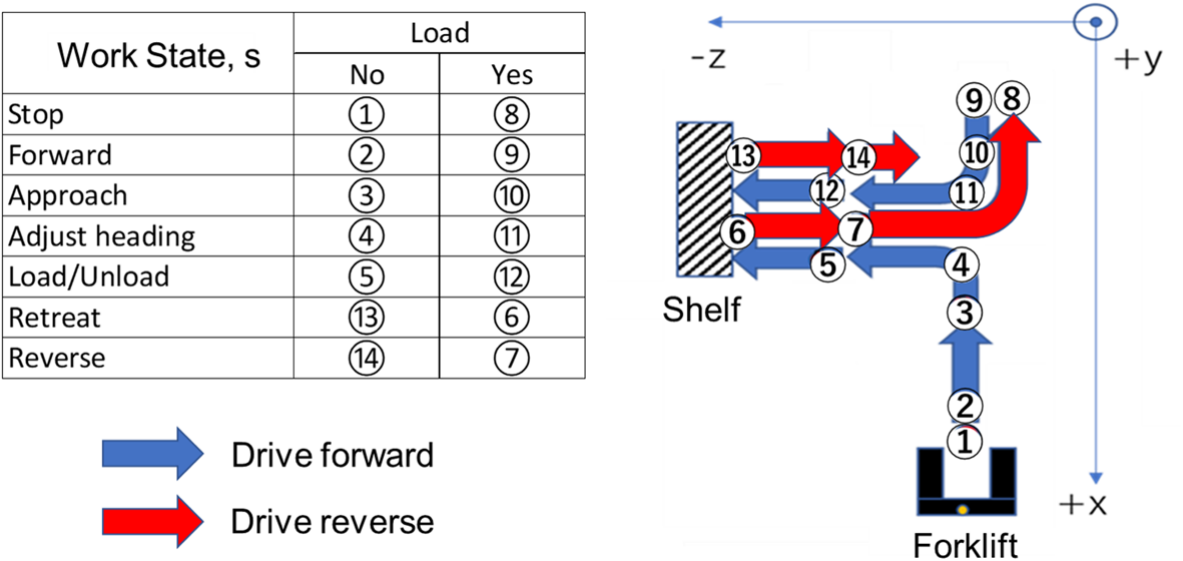
A typical forklift operation consists of a sequence of basic work states, where a comple cycle consists of 14 states.

D_4_ is an adaptiv﻿e view which appears only for complex operations. Referring to Fig. 6, these operations are cargo handling at work states ⑤ and ⑫, and reversing the forklift at work states ⑥, ⑦, ⑬, and ⑭. These operations are defined as complex because they require multiple salient attention.

For an ideal adaptive attention-based teleoperation system, operator is expected to focus on adaptive HMI elements D_1_, D_2_, and D_4_, where the optimal views are defined by outputs of camera selection method Y_1_, Y_2_, and Y_3_, respectively.

### Work state transition

From this section onwards, the method of computing the optimal visual stimuli Y_1_, Y_2_, and Y_3_ for HMI elements is elaborated. The adaptability of the HMI is supported by the ability of the system to recognize basic work states of forklift operation. In this case, the operation task defined in Fig. 2 is segmented into 14 basic work states which are typical of any forklift operations (see Fig. 6 which illustrates a cycle of basic work states). This approach is adapted from the preceding study^8^ which recognizes 6 basic work states. In the current study, the model is expanded to recognize 14 basic work states, thus enabling the model to recognize typical forklift work using higher resolution.

In Fig. 3, input of the work state model is the operation input vector **u** = (a_in_, θ_ty_, L_in_, R_in_, T_in_). Each dimension of **u** is a normalized voltage value measured from the potentiometer of the forklift’s operation levers. The first element a_in_ represents input from the acceleration lever which implicitly represent the linear velocity of the forklift’s drive wheel. The angle of this drive wheel is given by the second element θ_ty_. The other three elements L_in_, R_in_, and T_in_, represent inputs from the handling levers which control lift, reach and tilt of the forklift, respectively.

**Figure 7:**
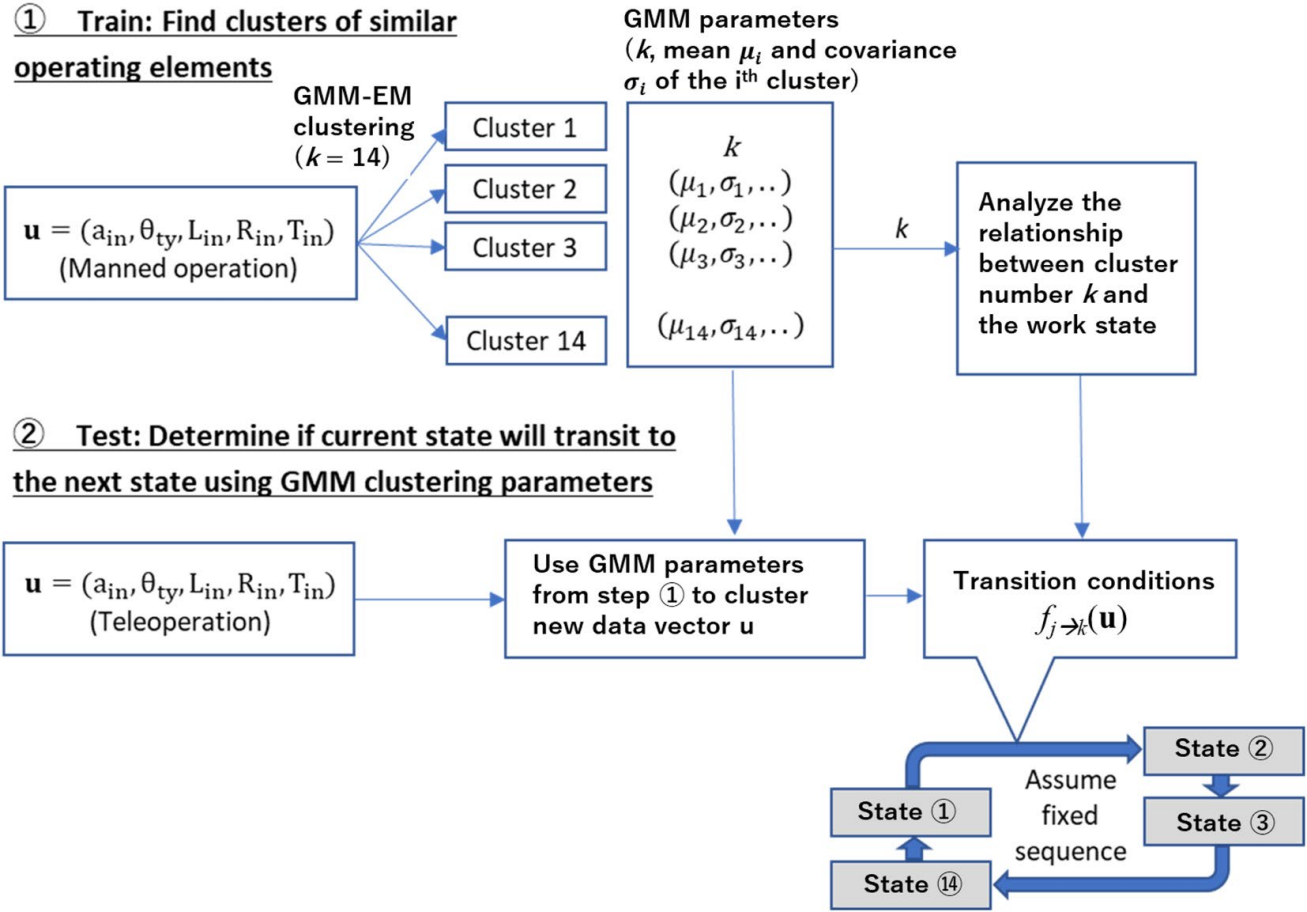
Work state estimation approach consists of training and testing of the GMM model, and using the parameters as transition conditions.

The output of work state model is work state s ∈ [1,14]. This model assumes the typical work state cycle in Fig. 6 and determines the probability of transition from work state at time t to t + 1 using parameters of **u** and its corresponding Gaussian Mixture Model (GMM). This process is illustrated in Fig. 7, where the first step uses data of manned forklift operation to train a GMM, and the parameters are used in the second step to determine work state transition using **u** of teleoperation.

Given X_t_ ∈ [1,14], the probability of transition from work state at time t to t + 1 is given by (1) and (2), where *f*_*j→k*_ is the transition condition from work state at time t to t + 1. Thus, given the initial work state X_t=0_ = 1 for a typical forklift operation cycle, the work state transition can be estimated sequentially by checking **u**_t_ at every sampling instant t during teleoperation.

1 $$P\left( {{\text{X}}_{{{\text{t}} + 1}} = {\text{k}}|{\text{X}}_{{\text{t}}} = {\text{j}}} \right) = {\text{p}}_{{{\text{jk}}}}$$

2 $${\text{p}}_{{{\text{jk}}}} = f_{j \to k} \left( {{\mathbf{u}}_{{\text{t}}} } \right),\;{\text{p}}_{{{\text{jk}}}} \in \left\{ {0,1} \right\}$$

### Gaze attention

The optimal visual stimuli for each work state are selected by referring to operators’ gaze attention during manned forklift operation as in Assumption 2. In the preceding study^9^, spatial analysis of point pattern was used to evaluate differences of gaze fixation pattern between different categories of operators and between different work states. The results suggest that major gaze fixations of different categories of operators at each work state are similar, and the common gaze fixations at each work state for these operators can be modeled by hierarchical clustering of their gaze fixations. More importantly, the common gaze fixations at each work state are representative of gaze fixations of all categories of operators as evaluated by their significant spatial correlations. However, the results show spatial independence between common gaze fixations for different work states especially for those after loading due to view occlusions by the cargo.

Therefore, the results of spatial analysis from the preceding study^9^ led to Assumption 2, and the common gaze attention **v** for work state s is defined by (3), which is a set of gaze points **g** ∈ ℝ^3^ and N_i_ is the number of gaze points for the ith work state.

3 $${\mathbf{v}}({\mathbf{s}}) = \left\{ {\begin{array}{*{20}c} {\left\{ {{\mathbf{g}}_{{\mathbf{1}}} ,{\mathbf{g}}_{{\mathbf{2}}} , \ldots ,{\mathbf{g}}_{{{\mathbf{N}}_{{\mathbf{1}}} }} } \right\},\;{\text{s}} = 1} \\ {\left\{ {{\mathbf{g}}_{{\mathbf{1}}} ,{\mathbf{g}}_{{\mathbf{2}}} , \ldots ,{\mathbf{g}}_{{{\mathbf{N}}_{{\mathbf{2}}} }} } \right\},\;{\text{s}} = 2} \\ \vdots \\ {\left\{ {{\mathbf{g}}_{{\mathbf{1}}} ,{\mathbf{g}}_{{\mathbf{2}}} , \ldots ,{\mathbf{g}}_{{{\mathbf{N}}_{{{\mathbf{14}}}} }} } \right\},\;{\text{s}} = 14} \\ \end{array} } \right.$$

**Figure 8:**
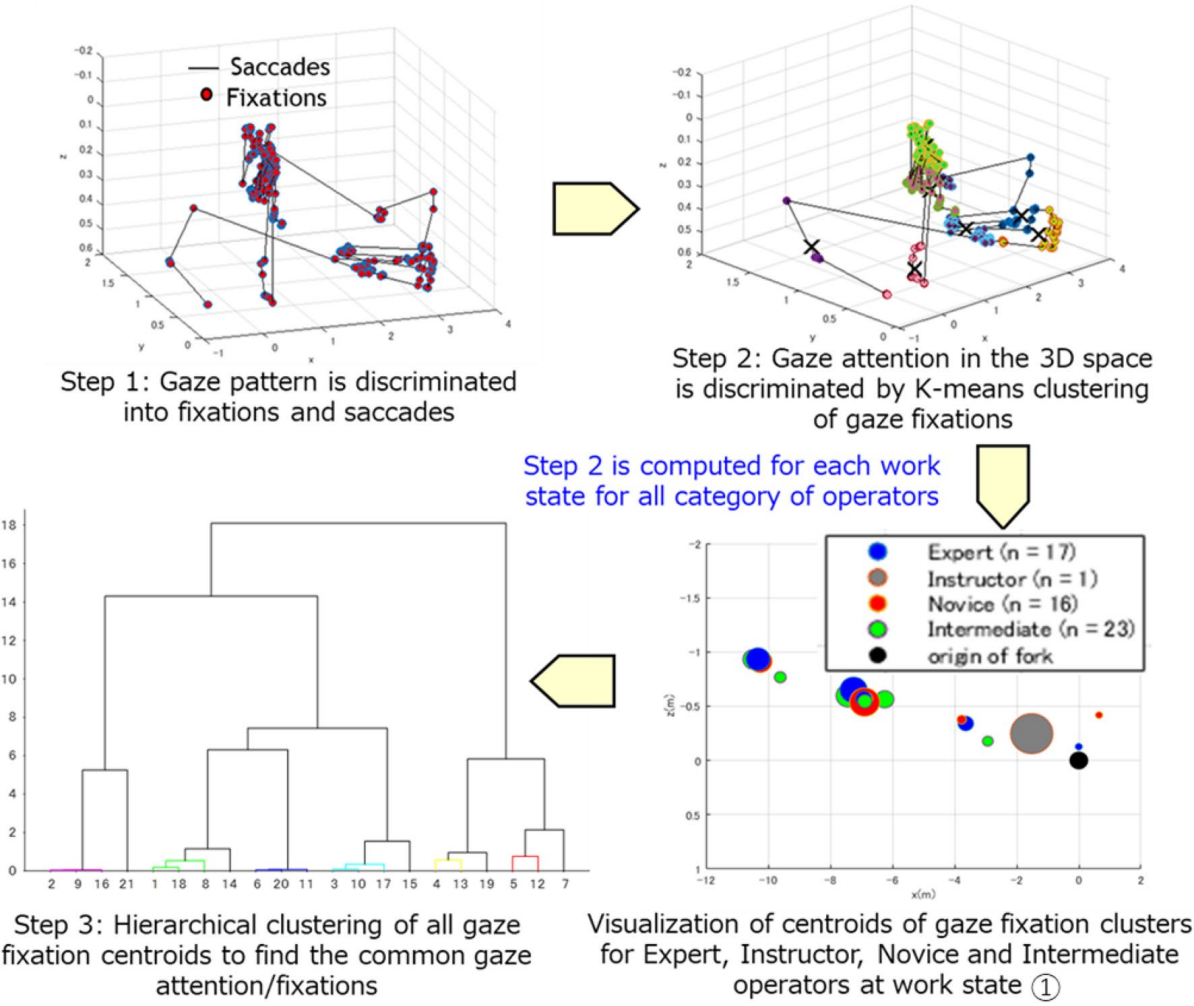
Gaze analysis approach to find common gaze attention (fixations) of operators from different categories.

The analysis of operators’ gaze fixations is based on the concept of foveal and peripheral vision^9,10^. It is noteworthy that visual recognition of human is not limited to the gaze fixation point itself. Instead, human recognize the fovea and parafovea vision areas which are the surrounding area of a fixation point. Thus, this spherical-like visual recognition area can be well-represented using the clustering approach. The analytic approach for gaze attention is summarized in Fig. 8. The analysis mainly focuses on clustering gaze fixation points because this stationary gaze pattern is more relevant to information processing and decision making.

In Fig. 8, gaze positions of each category of operators are first discriminated into fixations and saccades for each work state. Then, gaze fixations are clustered into several clusters using K-means clustering. The optimal K is selected using the silhouette plot^9,11^ and the elbow method^9,12^. The first and second steps are carried out for each category of operators at each work state. For the adaptive teleoperation HMI easy-to-use for different category of operators, the similarity of gaze fixations between different category of operators is evaluated using hierarchical clustering in the third step. The common gaze fixations between different category of operators are denoted as **g** in (3), where N_i_ denotes the number of common gaze fixations for the i^th^ work state.

### Selection of adaptive visual stimuli

**Figure 9:**
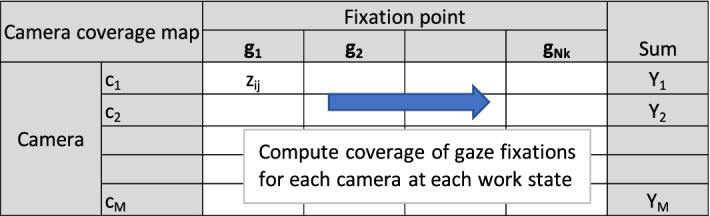
Illustration of camera coverage of gaze fixations at a work state.

This section explains the method for finding the optimal visual stimuli Y_1_, Y_2_, and Y_3_, for adaptive HMI elements, where Y_1_, Y_2_, Y_3_ ∈ {c_1_, c_2_, … , c_M_} and c_i_ is the view from the i^th^ camera mounted on the forklift. In this study, M = 8 and the positions of each camera are illustrated in Appendix B. To select the optimal visual stimuli, camera coverage of a set of gaze fixation points **v(s)** is computed based on^13^. The model of a camera is given by **C** = (X_c_, Y_c_, Z_c_, P, T, ccd_w_, ccd_h_, f), where (X_c_, Y_c_, Z_c_) is the position of the camera’s optical center, (P,T) is the yaw and pitch angles, and (ccd_w_, ccd_h_, f) is width, height, and focal length of the imaging plane. The concept is to evaluate the visibility of a fixation point on a camera’s image plane as in (4) and (5). Given a gaze fixation point g(x_g_, y_g_, z_g_), its projection on the imaging plane (x,y) of a camera positioned at (X_c_, Y_c_, Z_c_), with yaw and pitch angles (P,T) is defined by (4). The focal length and scale factor are represented by f and λ, respectively. The visibility of **g** on the image plane (x,y) can be computed by (5) (see Fig. 9).

4 $$\lambda \left[ {\begin{array}{*{20}c} f \\ x \\ y \\ \end{array} } \right] = \left[ {\begin{array}{*{20}c} {\cos T} & 0 & { - \sin T} \\ 0 & 1 & 0 \\ {\sin T} & 0 & {\cos T} \\ \end{array} } \right]\left[ {\begin{array}{*{20}c} {\cos P} & {\sin P} & 0 \\ { - \sin P} & {\cos P} & 0 \\ 0 & 0 & 1 \\ \end{array} } \right]\left[ {\begin{array}{*{20}c} {x_{g} - X_{C} } \\ {y_{g} - Y_{C} } \\ {z_{g} - Z_{C} } \\ \end{array} } \right]$$

5 $$z_{ij} = \left\{ {\begin{array}{*{20}c} {1,} & {x \in \left[ { - \frac{{ccd_{w} }}{2},\frac{{ccd_{w} }}{2}} \right],y \in \left[ { - \frac{{ccd_{h} }}{2},\frac{{ccd_{h} }}{2}} \right]} \\ {0,} & {x \notin \left[ { - \frac{{ccd_{w} }}{2},\frac{{ccd_{w} }}{2}} \right],y \notin \left[ { - \frac{{ccd_{h} }}{2},\frac{{ccd_{h} }}{2}} \right]} \\ \end{array} } \right.$$

Based on (4) and (5), camera coverage of all gaze fixations for each work state can be computed as illustrated in Fig. 9. Equation (6) computes camera coverage Y_i_ for the i^th^ camera for N_k_ gaze fixations at the k^th^ work state. Intuitively, Y_i_ is simply a measure of how many gaze fixations are seen by the i^th^ camera. Thus, the highest and second highest Y_i_ are assigned to D_1_ and D_2_, respectively, as the “Focused” views. The third highest Y_i_ is assigned to D_4_ as the “Alerting” view.

6 $${\text{Y}}_{{\text{i}}} = \sum\limits_{{\text{j}}}^{{{\text{N}}_{{\text{k}}} }} {{\text{z}}_{{{\text{ij}}}} }$$

### Usability test

A usability test is carried out to test the proposed adaptive teleoperation HMI described in the preceding sections. The proposed system is benchmarked with two other teleoperation HMIs. All the three teleoperation HMIs are briefly described below (the main difference is the visual stimuli presented on HMI elements D_1_ and D_2_ as illustrated in Fig. 1).

**Figure 10:**
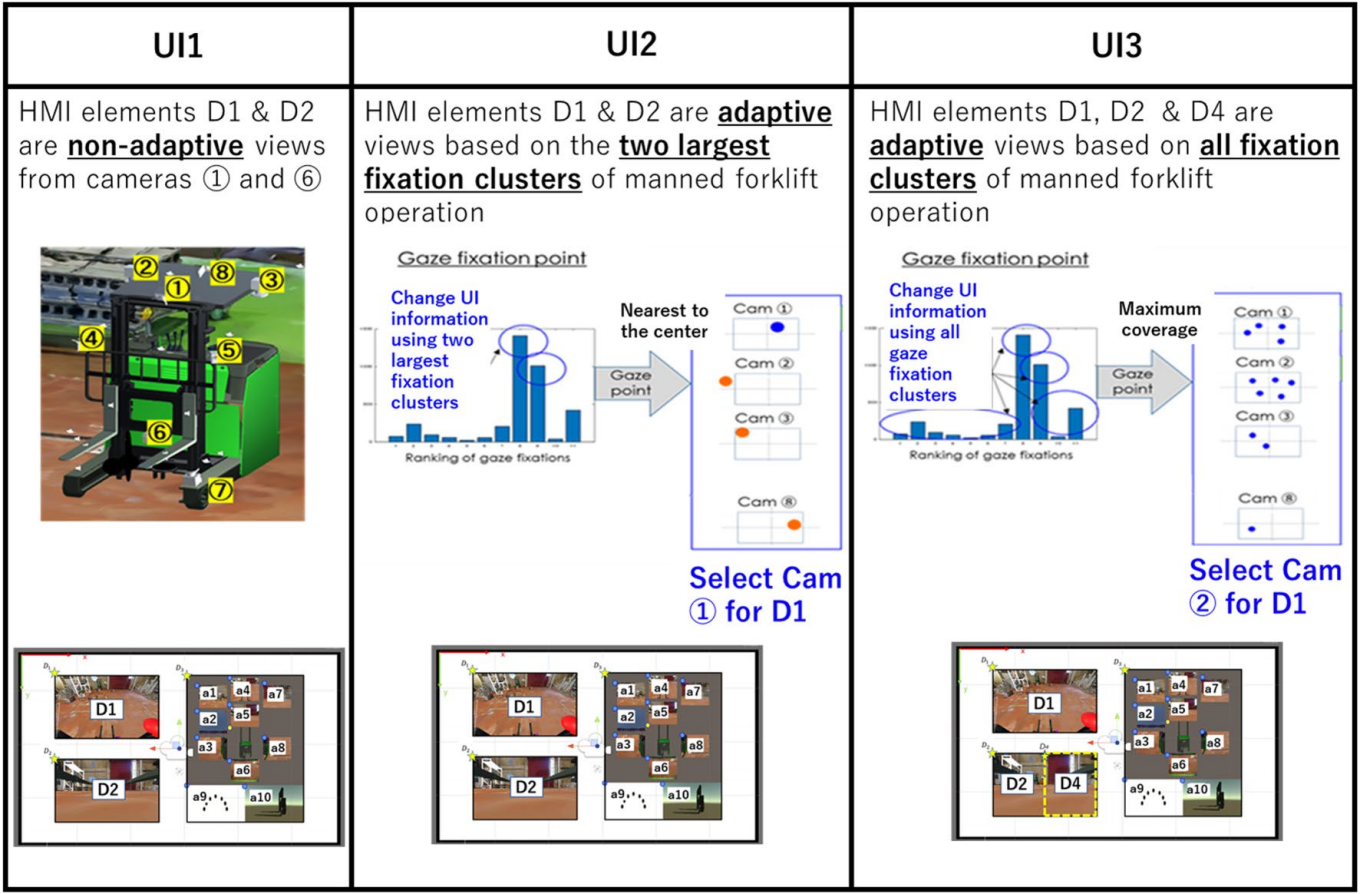
Illustration of the differences between HMI candidates for the usability test.

- UI1: Visual stimuli presented on D_1_, D_2_ are non-adaptive, i.e. fixed visual stimuli are presented like typical teleoperation HMI
- UI2: AVS are presented on D_1_, D_2_, but the method of selecting AVS is different from Fig. 9. Instead of using camera coverage map, AVS for D_1_ and D_2_ use only the two most frequently used gaze fixations at each work state (see Fig. 10)
- UI3: As described in the preceding sections (see Fig. 9)

This usability test is participated by two groups of new subjects, i.e. 14 Expert and 15 Beginner of manned forklift operation. They performed the task specified in Fig. 2 repeatedly in a pre-defined sequence to reduce effect of adaptation/learning. Instead of using across-subjects counterbalancing, this study uses within-subject counterbalancing to minimize the order effect^14^. Each subject carried out one training using UI3, followed by six tests in the following sequence (UI2 → UI1 → UI3 → UI3 → UI1 → UI2). Each UI is presented more than once but equally often for every subject in the opposite sequence. Therefore, the progressive error due to order effect can be cancelled/averaged out for every subject. The preference for within-subject counterbalancing is due to the difficulties of presenting the many possible orders equally and randomly to every subject. This method also averages out the adaptation to forklift teleoperation arising from repeated trials. After each test, subjects answered the NASA-Task Load Index (NASA-TLX) questionnaire and made pairwise comparison between the latest test and the test which was perceived to be the best.

## Results

During the usability test, each HMI was tested twice, and subjects were interviewed and asked to select the better HMI for teleoperation. The results of this interview are cross-checked with the operation time and perceived workload induced by each HMI. The preferred HMI is expected to complete the operation task in the shortest duration, and induce the lowest perceived workload (i.e. lowest NASA-TLX score).

### User feedback

Subjects were interviewed after each test, where they indicated their preference by comparing the most preferred HMI with the latest test which they have just performed. For example, the first comparison is made between Training and Test1, then the preferred HMI is used for comparison with Test2. This continued until the completion of Test6, where the preferred HMI is defined as the one which is perceived as the best for teleoperation of forklift. Table 1 shows the results of interview, where 2 Expert and 1 Beginner perceived UI1 and UI3 to be similar. The overall and categorial results for Expert and Beginner indicate that subjects perceived UI3 to be the best (62.5% to 68.8%), followed by UI1 (31.3%) and UI2 (0.0% to 6.3%). The result of the interview is consistent across different categories of subjects.

**Table 1:** Subjective perception of HMI.

	All (n = 29)		Expert (n = 14)		Beginner (n = 15)	
	n	%	n	%	n	%
UI1	10	31.3	5	31.3	5	31.3
UI2	1	3.1	0	0.0	1	6.3
UI3	21	65.6	11	68.8	10	62.5
Total	32	100.0	16	100.0	16	100.0

### Operation time

**Figure 11:**
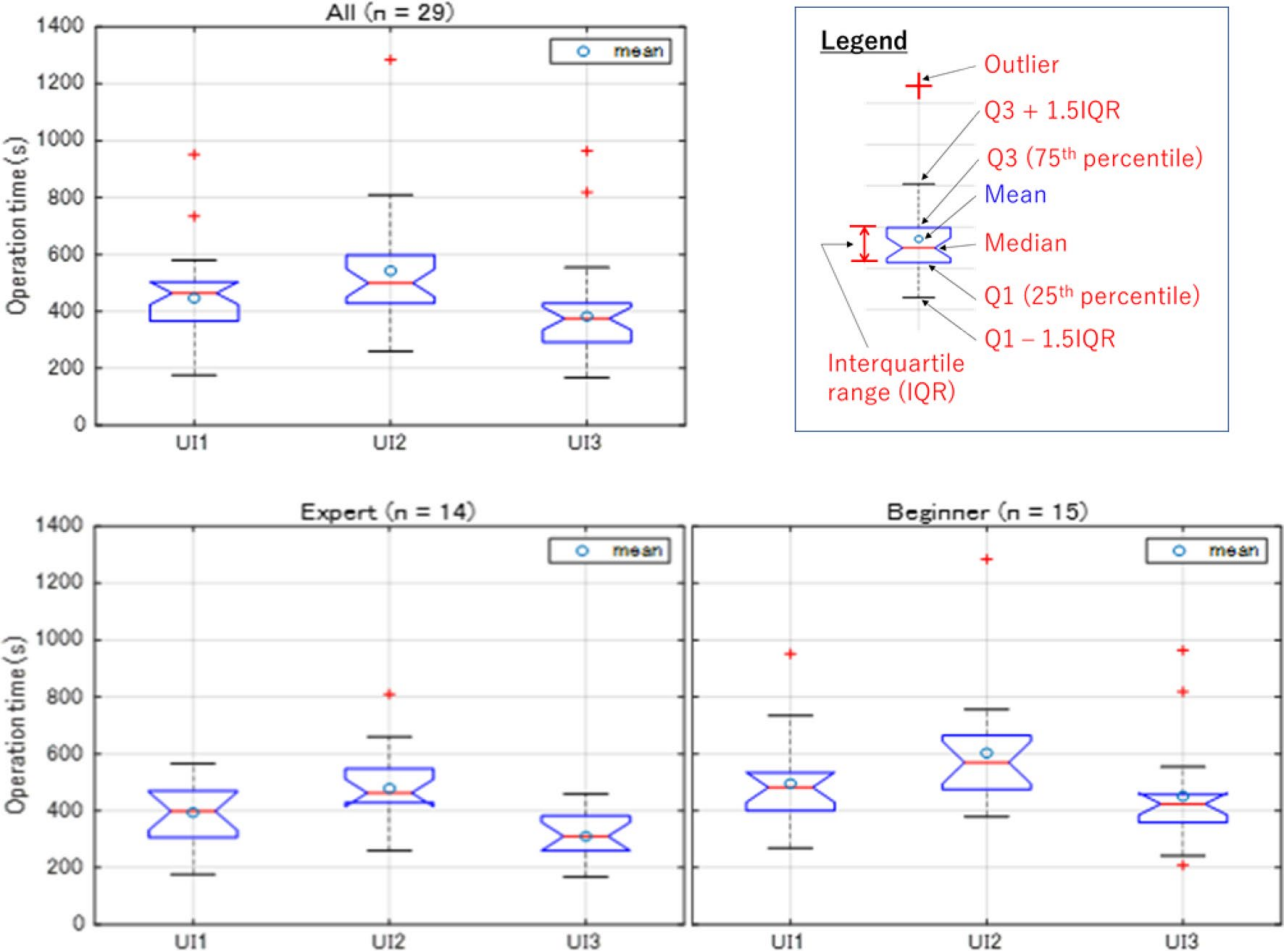
Operation time of each HMI.

The average operation time for each HMI is summarized in Fig. 11, where each boxplot summarizes the mean, median, 25th and 75th percentile of this response. Comparisons are made between UI1, UI2, and UI3 for different category of subjects, where subjects consistently completed the task in the shortest time using UI3, followed by UI1 and UI2. This result is consistent with the feedback of interview where most subjects perceived UI3 and UI2, as the most preferred and least preferred HMI for teleoperation, respectively. In other words, the preference of subjects from the interview is likely to be dependent on the time spent on completing the task. The operation time differences between UI1, UI2, and UI3, are generally statistically significant (*p* < 0.05) as in Table 2. The Shapiro–Wilk normality test is used to test the normality of the response data prior to selecting either the parametric or nonparametric tests to test the differences between dependent samples.

**Table 2:** Statistical analyses for operation time.

Operation time (s)		All (n = 29)			Expert (n = 14)			Beginner (n = 15)		
		UI1 versus UI2	UI1 versus UI3	UI2 versus UI3	UI1 versus UI2	UI1 versus UI3	UI2 versus UI3	UI1 versus UI2	UI1 versus UI3	UI2 versus UI3
Shapiro–Wilk (*p* > 0.05)	*p* value	0.0009	0.0265	0.0005	0.1564	0.0423	0.1035	0.0155	0.0116	0.0169
	Normality	0	0	0	1	0	1	0	0	0
Paired t-test	t-stat	− 3.2145	3.8466	4.4476	− 3.0663	4.6534	5.9470	− 2.0283	1.6602	2.3177
	*p* value	0.0033	0.0006	0.0001	0.0090	0.0005	0.0000	0.0620	0.1191	0.0361
Wilcoxon signed rank	z-value	− 3.2975	3.4489	3.9895	− 2.8759	3.8419	3.8419	− 2.0409	1.9160	2.4991
	*p* value	0.0010	0.0006	0.0001	0.0040	0.0001	0.0001	0.0413	0.0554	0.0125
Statistical significance		1	1	1	1	1	1	1	0	1

In this study, the former and latter refers to the paired t-test and the Wilcoxon Signed Rank test, respectively. The nonparametric test is used when the null hypothesis of Shapiro–Wilk normality test is rejected at *p* < 0.05. This means the null hypothesis which assumes the distribution of data as normally distributed is rejected. Additional analyses data such as the skewness of the distribution of data can be found in Appendix C. Due to the presence of outliers as indicated by boxplots in Fig. 11, the response data are generally skewed. Therefore, statistical significance of the differences of operation time between different HMIs is mostly tested using the Wilcoxon Signed Rank test which is more robust to outliers. It is also noteworthy that test results using either the parametric or nonparametric tests are almost consistent, except for Beginner.

### Perceived workload

**Figure 12:**
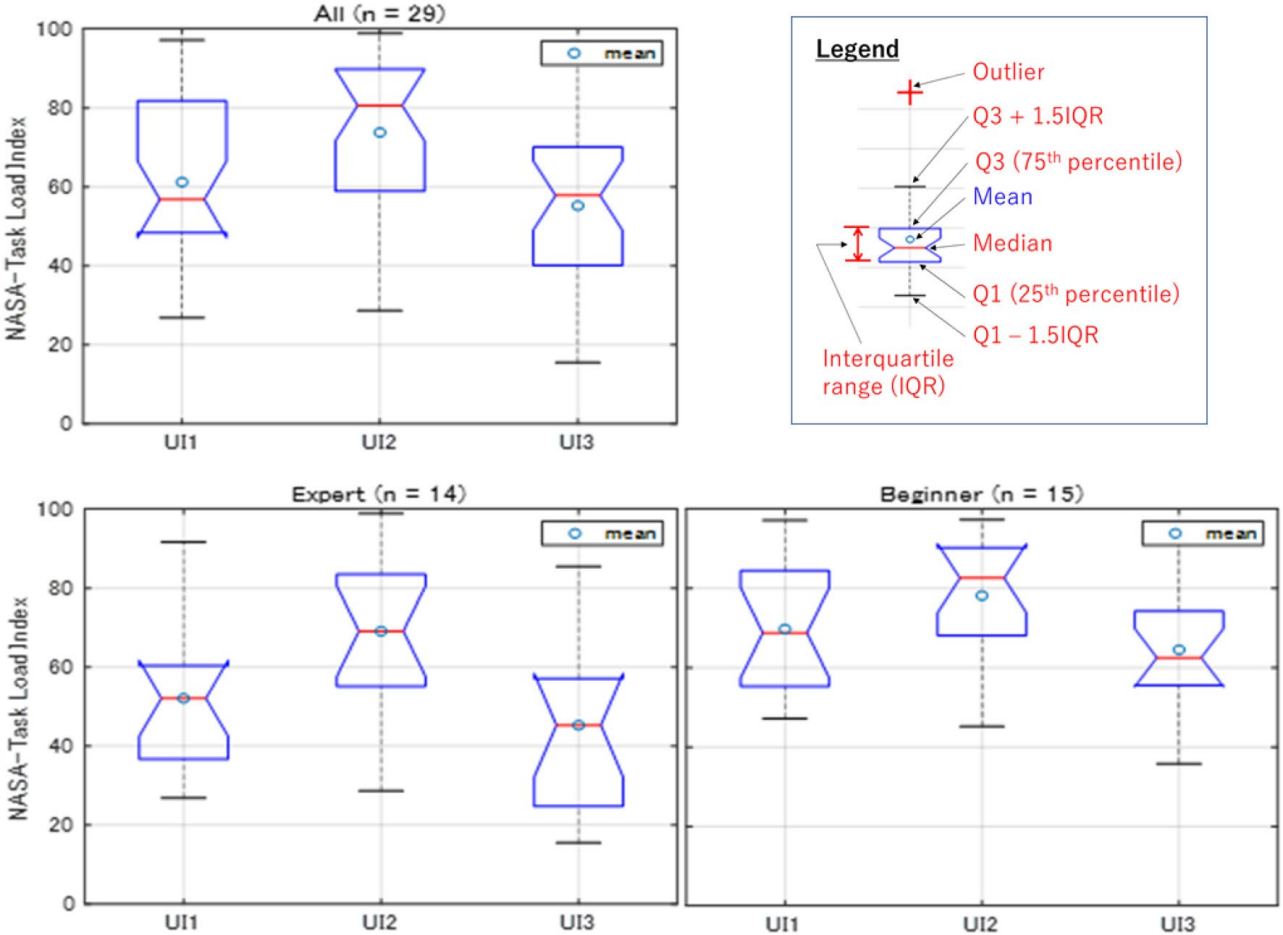
NASA-TLX (workload) of each HMI.

The results of perceived workload (NASA-TLX) are also consistent with the results of subjective preference in Table 1, where UI3 is preferred regardless of the category/skills of subjects, The results of NASA-TLX for operators performing the task using different HMI is summarized in Table 3 and Fig. 12. The perceived workload is consistently the lowest for UI3, followed by UI1, and UI2. This means the preference of subjects is also influenced by the perceived workload when using different HMIs for teleoperation. In other words, the subjective preference of subjects is likely to be influenced by operation time and perceived workload, where the responses are consistent with each other. Table 3 shows the statistical tests for the differences of NASA-TLX for different HMIs. The test is carried out like the preceding section by checking the normality of the response data prior to selecting either the paired t-test or Wilcoxon Signed Rank test. Results indicate the differences of NASA-TLX are also generally statistically significant (*p* < 0.05) except for Beginner (see Appendix C for the complete test result).

**Table 3:** Statistical analyses for perceived workload (NASA-TLX).

Perceived workload (NASA-TLX)		All (n = 29)			Expert (n = 14)			Beginner (n = 15)		
		UI1 versus UI2	UI1 versus UI3	UI2 versus UI3	UI1 versus UI2	UI1 versus UI3	UI2 versus UI3	UI1 versus UI2	UI1 versus UI3	UI2 versus UI3
Shapiro–Wilk (*p* > 0.05)	*p* value	0.0600	0.5355	0.0311	0.0951	0.6790	0.1714	0.3722	0.2855	0.0535
	Normality	1	1	0	1	1	1	1	1	1
Paired t-test	t-stat	− 4.0252	2.8599	5.3598	− 4.0222	2.0679	4.5831	− 1.9011	1.9109	3.0994
	*p* value	0.0004	0.0079	0.0000	0.0015	0.0592	0.0005	0.0781	0.0767	0.0078
Wilcoxon signed rank	z-value	− 3.3516	2.9194	4.1843	− 3.3345	2.3214	3.8419	− 1.6735	1.9781	2.4306
	*p* value	0.0008	0.0035	0.0000	0.0009	0.0195	0.0001	0.0942	0.0498	0.0157
Statistical significance		1	1	1	1	0	1	0	0	1

## Discussions

### Factors of NASA-TLX

**Figure 13:**
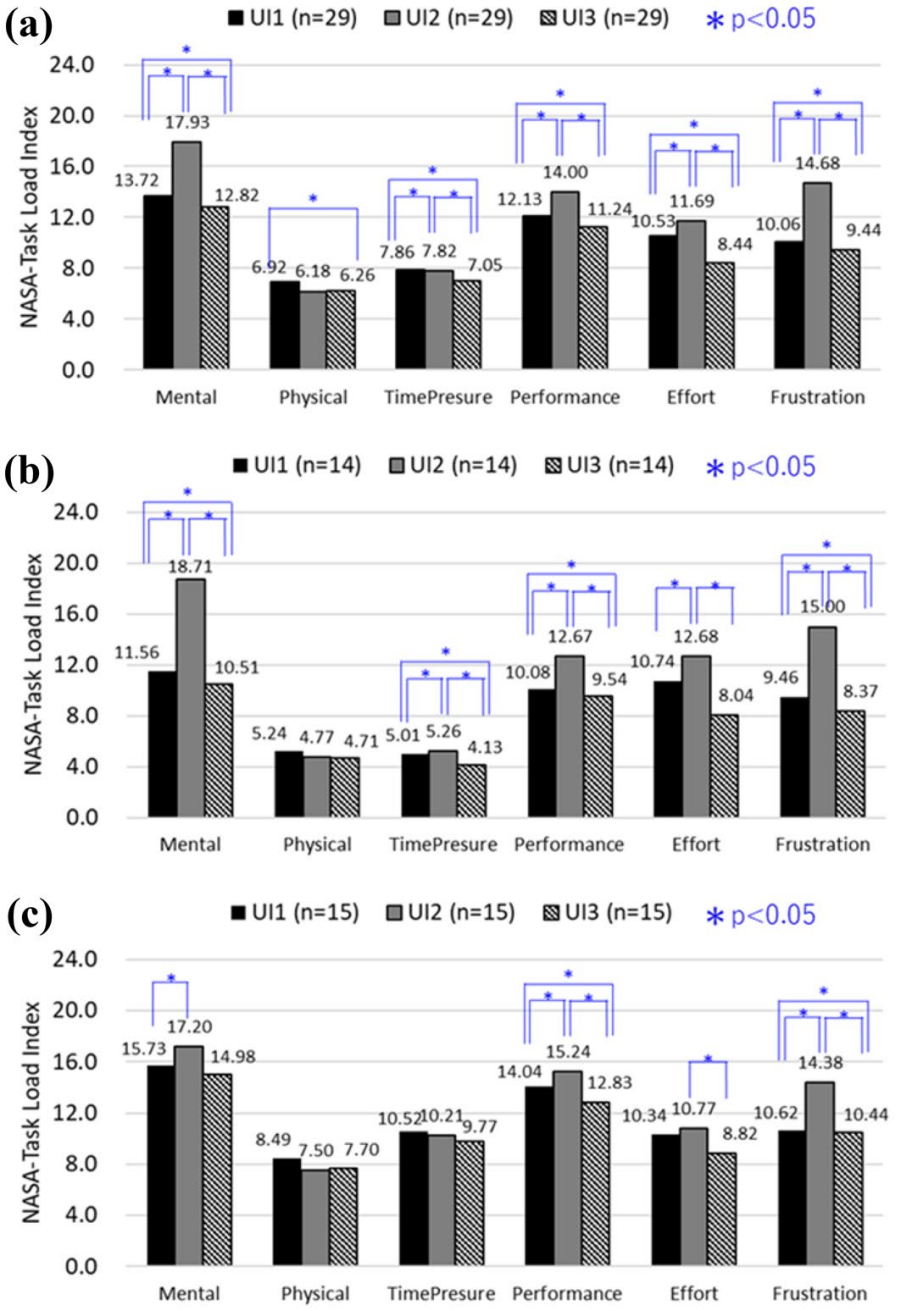
Weighted NASA-TLX responses for (**a**) all subjects, (**b**) experts, and (**c**) beginners.

The weighted NASA-TLX^15^ is used to evaluate the workload of subjects so that the factors which are relevant to the experiment task can be evaluated. There are six factors, i.e. Mental, Physical, Time Pressure, Performance, Effort and Frustration, and they are weighted using pairwise comparisons between each other. In total, subjects made 15 pairwise comparisons and the resulting scoring are used as weights to compute the weighted NASA-TLX. The breakdown of weighted NASA-TLX responses for six factors is summarized in Fig. 13, and analyses results of normality tests and dependent sample tests are tabulated in Appendix C.

Except for the factors Physical and Time Pressure, every factor exhibits similar scoring pattern in all cases, i.e. the score is the lowest for UI3, followed by UI1, and UI2. The differences of responses between UIs are generally statistically significant (*p* < 0.05) as indicated in Fig. 13a. This means, subjects perceived UI3 is better than UI1 and UI2 in the following aspects (factors), i.e. Mental, Performance, Effort and Frustration. This perception is consistent with the significantly lower operation time as indicated in Fig. 11. For factors Physical and Time Pressure, the scoring pattern is inconsistent and mostly statistically insignificant compared to other factors, especially for Beginners. This is maybe because these two factors are less related to the task which requires insignificant physical movement, and the task had no time limit. The more evident inconsistency for Beginners is reasonable since Beginners usually exhibit higher variances. For example, previous studies have indicated responses of novice swimmers^16^ and crane operators^17^, consist of higher standard deviations.

Apart from the inconsistency for factors Physical and Time Pressure, responses of Expert and Beginner for other factors are consistent as shown in Fig. 13b,c. However, responses of Expert are lower than that of Beginner for these factors, specifically for UI3 which is perceived to be the best for teleoperation. This is reasonable considering that subjects of the Expert category are more likely to quickly adapt to teleoperation, so the workload tends to be lower across the different NASA-TLX factors. However, the results suggest this is true only for UI3 which is perceived to be the best for remote operation. On the contrary, UI2 which is perceived to be the worst for teleoperation, prompted Expert to score higher than Beginner for factors such as Mental, Effort, and Frustration. This maybe because Expert subjects may find UI2 more difficult to use because they have at least some expectations on the optimal visual stimuli due to their prior knowledge. Inappropriate visual stimuli presented by UI2 may therefore prompt Expert subjects to score higher compared to Beginner subjects who have relatively less prior knowledge.

### Fixed versus adaptive visual stimuli

Referring to Fig. 13, the Physical factor is consistently the largest for UI1 compared to UI2 and UI3. This response is different compared to the other factors which consistently show UI2 as the largest. This means subjects perceived HMIs with AVS (i.e. UI2, UI3) to require lower physical load compared to HMI with fixed visual stimuli HMI (i.e. UI1). This seems to be reasonable considering that subjects do not need to frequently move the eyes and heads to search for optimal visual stimuli, which will be automatically shown on adaptive HMI elements for UI2 and UI3. In other words, teleoperation HMI with AVS reduces the burden of subjects by providing optimal visual stimuli at each work state on predefined HMI elements. This makes the adaptive HMI system to be better than the fixed information HMI system, where subjects need to think and search for the optimal visual stimuli.

The preference for AVS for teleoperation system can be traced to the trend in manned operation systems. Increasing sensing capability using wide angle cameras like fisheye^18^ and omnidirectional^19^ cameras provide rich visual information to operators so that it is no longer necessary to search for the desired visual information. Coupled with the improvement in computing power and cutting-edge algorithms for computer vision and machine learning, rich visual information can be processed quickly to facilitate operations of autonomous or manned systems. Therefore, in the case of semi-autonomous operations, it is important to have a support system to present the optimal visual stimuli at the appropriate timing, especially for teleoperation of multiple vehicles.

The proposed AVS is promising for such purpose because it is developed based on behavioral data of human operators. The advantage of biological intelligence was discussed by^20^. Compared to related studies on AVS,^21,22^ proposed using views of autonomous monitoring robots for teleoperation,^23^ proposed using real-time manipulation of camera, and^24^ proposed real-time 3D reconstruction of environment. Therefore, the proposed system based on behavioral data of human operators is advantageous because it does not require additional supporting systems like monitoring robots or camera manipulation system, or real-time 3D reconstruction of the environment that requires high computing power.

### Responses of expert versus beginner

It is noteworthy that both Expert and Beginner subjects exhibit consistent responses which indicate UI3 is relatively the best HMI for teleoperation of forklift. This suggests that UI3 is easy to use for both category of subjects. This similarity of responses maybe because both Expert and Beginner for manned forklift operation are novices for teleoperation.

Referring to Fig. 11, Experts tend to complete the task in shorter duration compared to Beginners, which is consistent for every HMI. The corresponding perceived workload NASA-TLX is shown in Fig. 12, and both illustrate the same pattern. Specifically, Experts exhibit lower perceived workload compared to Beginners. The results suggest shorter operation time translates into lower perceived workload, and the vice versa. Normality tests have indicated the distribution of operation time and perceived workload is skewed and normal, respectively. Therefore, the Mann–Whitney U-test (Wilcoxon rank sum test) and the two samples t-test are used to analyze the differences of operation time and perceived workload, respectively. The results are tabulated in Table 4.

**Table 4:** Comparison of responses between expert and beginner.

	Expert (n = 14) versus beginner (n = 15)		
	UI1	UI2	UI3
Operation time			
Significance	0	0	1
*p* value	0.1112	0.1017	0.0154
z-value	− 1.5930	− 1.6366	− 2.4222
Ranksum	173	172	154
NASA-TLX			
Significance	1	0	1
*p* value	0.0173	0.1961	0.0152
t-stat	− 2.5368	− 1.3256	− 2.5923
Degree-of-freedom	27	27	27
SD	18.7335	18.4829	19.9791

The differences between Experts and Beginners are statistically significant at *p* < 0.05 for UI3, for both operation time and perceived workload. For UI2, both the differences are not statistically significant, and for UI1, only the difference of perceived workload is statistically significant. The result is reasonable considering that UI3 is the most preferred UI that facilitates teleoperation of forklift. In case of UI1 and UI2, the responses have higher variances since non-optimal visual stimuli were presented during the experiment. This means, given optimal visual stimuli at the appropriate timing like the case of UI3, statistically significant lower operation time and perceived workload can be achieved for forklift teleoperation using the proposed AVS4UI.

## Conclusions

The proposed adaptive attention-based HMI system addresses a critical issue in transitions from manned to teleoperation system. Human behavior models are used to bridge the gap between these two types of systems, where optimal visual stimuli are determined empirically rather than intuitively. Thus, it is possible to generalize this approach to develop teleoperation systems for different applications. Implementation of the proposed system in the simulation environment showed promising results, where the perceived workload is lower than that of non-adaptive HMI system. More importantly, the response is consistent for different category of operators which suggests the adaptive HMI system is easy-to-use.

However, it is noteworthy that humans are to be capable of understanding the current environment and acquire the desired visual stimuli in advance. This predictive ability is not yet embedded in the current system. As the future work, it is desirable to incorporate such predictive function and to implement the proposed system in the physical environment.

## Supplementary Information

Supplementary Information.
