# Supplementary material for: Adaptive attention-based human machine interface system for teleoperation of industrial vehicle
Source: Sci Rep. 2021 Aug 26;11:17284. doi: 10.1038/s41598-021-96682-0 (PMC8390500; doi:10.1038/s41598-021-96682-0)
Supplement: Supplementary file 1 — Supplementary Information. [file 41598_2021_96682_MOESM1_ESM.pdf]

Title:
Adaptive Attention-Based Human Machine Interface System for Teleoperation of Industrial Vehicle

Authors:
J.Y. Chew et al.

# Appendix A

Configuration of HMI elements (refer to Appendix B for camera details)

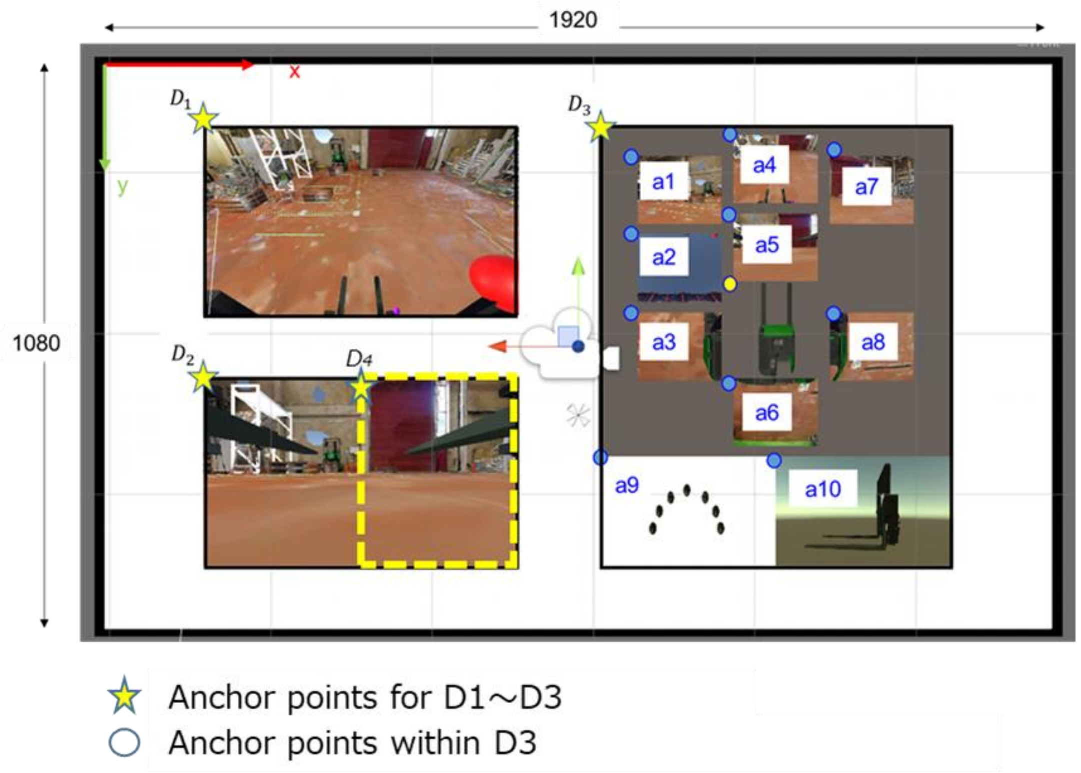


| Display | | Anchor point | | Resolution | Content |
|---|---|---|---|---|---|
| Main | Sub | x | y | | |
| D1 | | 200 | 120 | 640x360 | Algorithm - Camera ① to ⑧ |
| D2 | | 200 | 600 | 640x360 | Algorithm - Camera ① to ⑧ |
| D3 | | 1000 | 120 | 720x840 | Background for subdisplays |
| | a0 | 275 | 295 | 170x185 | Position of forklift image |
| | a1 | 80 | 55 | 170x130 | Camera ⑤—Front left |
| | a2 | 80 | 205 | 170x130 | Camera ⑦—Left leg |
| | a3 | 80 | 355 | 170x130 | Camera ③—Top left |
| | a4 | 275 | 15 | 170x130 | Camera ①—Front center |
| | a5 | 275 | 165 | 170x130 | Camera ⑥—Fork |
| | a6 | 275 | 480 | 170x130 | Camera ⑧—Back |
| | a7 | 470 | 55 | 170x130 | Camera ④—Front right |
| | a8 | 470 | 355 | 170x130 | Camera ②—Top right |
| | a9 | 1 | 630 | 360x210 | Tire Angle |
| | a10 | 360 | 630 | 360x210 | Tilt Angle |

Title:
Adaptive Attention-Based Human Machine Interface System for Teleoperation of Industrial Vehicle

Authors:
J.Y. Chew et al.

# Appendix B

Details of cameras mounted on the forklift

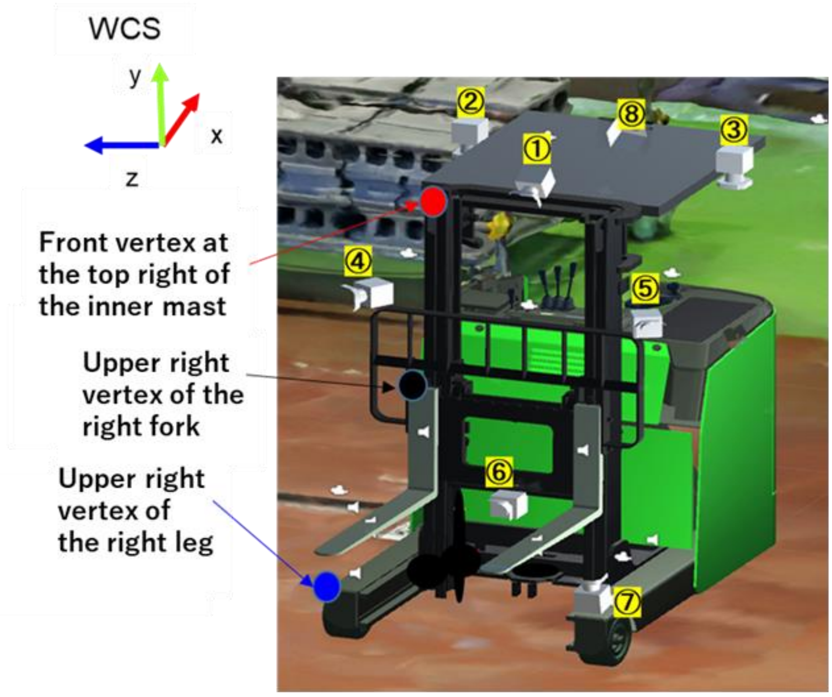


| Parameter | | Camera | | | | | | | |
|---|---|---|---|---|---|---|---|---|---|
| Specifications | | ① | ② | ③ | ④ | ⑤ | ⑥ | ⑦ | ⑧ |
| | FOV (°) | 80.0 | 60.0 | 60.0 | 60.0 | 60.0 | 60.0 | 60.0 | 60.0 |
| Position and orientation | | | | | | | | | |
| | x (m) | 0.10 | 1.10 | 1.10 | 0.34 | 0.34 | 0.29 | 0.03 | 1.70 |
| | y (m) | 0.19 | 2.00 | 2.00 | 0.56 | 0.56 | -0.59 | 0.00 | 2.00 |
| | z (m) | -0.33 | 0.15 | -1.22 | 0.14 | -0.86 | -0.36 | -1.00 | -0.54 |
| | $\phi$ (°) | 35.00 | 75.00 | 105.00 | 0.00 | 0.00 | 0.00 | -75.00 | 115.00 |
| | $\theta$ (°) | -90.00 | 0.00 | 0.00 | -60.00 | -120.00 | -90.00 | -90.00 | -90.00 |
| | $\varphi$ (°) | 0.00 | 0.00 | 0.00 | 0.00 | 0.00 | 0.00 | 0.00 | 0.00 |

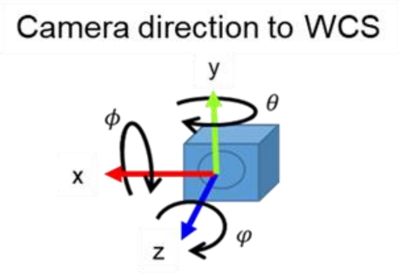

Title:
Adaptive Attention-Based Human Machine Interface System for Teleoperation of Industrial Vehicle

Authors:
J.Y. Chew et al.

# Appendix C

Statistical analyses using Shapiro-Wilk, Paired t-test, and Wilcoxon Signed Rank tests

| | | | All (n=29) | | | Expert (n=14) | | | Beginner (n=15) | | |
|---|---|---|---|---|---|---|---|---|---|---|---|
| | | | UI1 vs UI2 | UI1 vs UI3 | UI2 vs UI3 | UI1 vs UI2 | UI1 vs UI3 | UI2 vs UI3 | UI1 vs UI2 | UI1 vs UI3 | UI2 vs UI3 |
| NASA-TLX (Weighted Average Score) | Mean of differences | | 12.5747 | -5.9771 | -18.5517 | 16.9999 | -6.7978 | -23.7976 | 8.4445 | -5.2111 | -13.6555 |
| | Stdev of differences | | 16.8232 | 11.2546 | 18.6396 | 15.8142 | 12.3001 | 19.4283 | 17.2034 | 10.5614 | 17.0636 |
| | Median of differences | | 11.6670 | -5.6670 | -16.8330 | 14.9160 | -6.8335 | -21.2500 | 2.1670 | -1.6660 | -6.5000 |
| | Skewness | | 0.2826 | 0.0423 | -0.8507 | 0.5603 | 0.3684 | -0.9603 | 0.2981 | -0.3366 | -0.7885 |
| | Shapiro-Wilk | p-value | 0.0600 | 0.5355 | 0.0311 | 0.0951 | 0.6790 | 0.1714 | 0.3722 | 0.2855 | 0.0535 |
| | | normality | 1 | 1 | 0 | 1 | 1 | 1 | 1 | 1 | 1 |
| | Paired t-test | t-stat | -4.0252 | 2.8599 | 5.3598 | -4.0222 | 2.0679 | 4.5831 | -1.9011 | 1.9109 | 3.0994 |
| | | df | 28 | 28 | 28 | 13 | 13 | 13 | 14 | 14 | 14 |
| | | p-value | 0.0004 | 0.0079 | 0.0000 | 0.0015 | 0.0592 | 0.0005 | 0.0781 | 0.0767 | 0.0078 |
| | Wilcoxon Signed Rank | z-value | -3.3516 | 2.9194 | 4.1843 | -3.3345 | 2.3214 | 3.8419 | -1.6735 | 1.9781 | 2.4306 |
| | | signedrank | 49.5000 | 352.5000 | 411.0000 | 4.0000 | 89.0000 | 105.0000 | 21.0000 | 94.5000 | 101.5000 |
| | | p-value | 0.0008 | 0.0035 | 0.0000 | 0.0009 | 0.0195 | 0.0001 | 0.0942 | 0.0498 | 0.0157 |
| Operation time (s) | Mean of differences | | 96.7100 | -63.6114 | -160.3214 | 84.9971 | -83.9836 | -168.9807 | 107.6403 | -44.5990 | -152.2393 |
| | Stdev of differences | | 162.0174 | 89.0535 | 194.1160 | 103.7193 | 67.5291 | 106.3172 | 205.5369 | 104.0404 | 254.4035 |
| | Median of differences | | 70.9300 | -66.3900 | -137.5500 | 53.5300 | -60.6300 | -131.0750 | 72.2100 | -88.6040 | -137.5500 |
| | Skewness | | 1.7589 | 1.2903 | -0.9297 | 1.1820 | -0.6723 | -0.9026 | 1.5741 | 1.5411 | -0.9177 |
| | Shapiro-Wilk | p-value | 0.0009 | 0.0265 | 0.0005 | 0.1564 | 0.0423 | 0.1035 | 0.0155 | 0.0116 | 0.0169 |
| | | normality | 0 | 0 | 0 | 1 | 0 | 1 | 0 | 0 | 0 |
| | Paired t-test | t-stat | -3.2145 | 3.8466 | 4.4476 | -3.0663 | 4.6534 | 5.9470 | -2.0283 | 1.6602 | 2.3177 |
| | | df | 28 | 28 | 28 | 13 | 13 | 13 | 14 | 14 | 14 |
| | | p-value | 0.0033 | 0.0006 | 0.0001 | 0.0090 | 0.0005 | 0.0000 | 0.0620 | 0.1191 | 0.0361 |
| | Wilcoxon Signed Rank | z-value | -3.2975 | 3.4489 | 3.9895 | -2.8759 | 3.8419 | 3.8419 | -2.0409 | 1.9160 | 2.4991 |
| | | signedrank | 65.0000 | 377.0000 | 402.0000 | 9.0000 | 105.0000 | 105.0000 | 24.0000 | 94.0000 | 103.0000 |
| | | p-value | 0.0010 | 0.0006 | 0.0001 | 0.0040 | 0.0001 | 0.0001 | 0.0413 | 0.0554 | 0.0125 |
| NASA-TLX (Mental) | Mean of differences | | 13.2759 | -4.2241 | -17.5000 | 15.3571 | -9.6429 | -25.0000 | 11.3333 | 0.8333 | -10.5000 |
| | Stdev of differences | | 17.9048 | 12.2675 | 21.2447 | 21.9233 | 8.8174 | 23.2668 | 13.6561 | 13.1158 | 17.0660 |
| | Median of differences | | 10.0000 | -2.5000 | -17.5000 | 11.2500 | -8.7500 | -18.7500 | 7.5000 | 0.0000 | -2.5000 |
| | Skewness | | 0.9351 | 1.1916 | -0.7512 | 0.9078 | -0.2728 | -0.5865 | 0.3081 | 1.4470 | -0.5305 |
| | Shapiro-Wilk | p-value | 0.0530 | 0.0154 | 0.0656 | 0.0849 | 0.8767 | 0.3688 | 0.1250 | 0.0153 | 0.0204 |
| | | normality | 1 | 0 | 1 | 1 | 1 | 1 | 1 | 0 | 0 |
| | Paired t-test | t-stat | -3.9929 | 1.8543 | 4.4359 | -2.6210 | 4.0919 | 4.0204 | -3.2142 | -0.2461 | 2.3829 |
| | | df | 28 | 28 | 28 | 13 | 13 | 13 | 14 | 14 | 14 |
| | | p-value | 0.0004 | 0.0743 | 0.0001 | 0.0211 | 0.0013 | 0.0015 | 0.0062 | 0.8092 | 0.0319 |
| | Wilcoxon Signed Rank | z-value | -3.5153 | 2.2287 | 3.6020 | -2.4722 | 3.1366 | 2.9834 | -2.6017 | 0.1128 | 1.6257 |
| | | signedrank | 32.0000 | 195.0000 | 361.0000 | 11.0000 | 87.0000 | 100.5000 | 7.0000 | 23.5000 | 78.5000 |
| | | p-value | 0.0004 | 0.0258 | 0.0003 | 0.0142 | 0.0017 | 0.0011 | 0.0093 | 0.9414 | 0.1077 |
| NASA-TLX (Physical) | Mean of differences | | 2.9310 | -4.6552 | -7.5862 | 5.1786 | -5.8929 | -11.0714 | 0.8333 | -3.5000 | -4.3333 |
| | Stdev of differences | | 19.3657 | 9.5132 | 21.2078 | 20.8581 | 10.8578 | 24.0135 | 18.3387 | 8.2808 | 18.4552 |
| | Median of differences | | 0.0000 | -2.5000 | 0.0000 | -2.5000 | -5.0000 | -5.0000 | 0.0000 | -2.5000 | 0.0000 |
| | Skewness | | 0.7828 | -0.4983 | -0.6863 | 1.0457 | -0.4663 | -0.7697 | 0.4537 | -0.3146 | -0.2620 |
| | Shapiro-Wilk | p-value | 0.0309 | 0.1107 | 0.2512 | 0.0325 | 0.3393 | 0.4437 | 0.4223 | 0.3586 | 0.1507 |
| | | normality | 0 | 1 | 1 | 0 | 1 | 1 | 1 | 1 | 1 |
| | Paired t-test | t-stat | -0.8151 | 2.6352 | 1.9263 | -0.9290 | 2.0307 | 1.7251 | -0.1760 | 1.6370 | 0.9094 |
| | | df | 28 | 28 | 28 | 13 | 13 | 13 | 14 | 14 | 14 |
| | | p-value | 0.4219 | 0.0136 | 0.0643 | 0.3698 | 0.0633 | 0.1082 | 0.8628 | 0.1239 | 0.3785 |
| | Wilcoxon Signed Rank | z-value | -0.3291 | 2.5035 | 1.7597 | -0.1890 | 2.0336 | 1.4347 | -0.1135 | 1.4580 | 0.8772 |
| | | signedrank | 138.5000 | 220.0000 | 211.5000 | 36.0000 | 55.5000 | 57.5000 | 37.5000 | 58.0000 | 51.0000 |
| | | p-value | 0.7420 | 0.0123 | 0.0785 | 0.8350 | 0.0469 | 0.1577 | 0.9263 | 0.1465 | 0.3745 |
| NASA-TLX (Time Pressure) | Mean of differences | | 6.8966 | -6.4655 | -13.3621 | 10.0000 | -7.8571 | -17.8571 | 4.0000 | -5.1667 | -9.1667 |
| | Stdev of differences | | 18.0111 | 13.0848 | 18.6399 | 12.2474 | 9.0860 | 16.6658 | 22.1561 | 16.1871 | 19.9478 |
| | Median of differences | | 2.5000 | -2.5000 | -5.0000 | 2.5000 | -2.5000 | -17.5000 | 2.5000 | -5.0000 | 0.0000 |
| | Skewness | | -0.1666 | 0.1059 | -0.7175 | 1.1907 | -0.7971 | -0.7017 | -0.0625 | 0.0590 | -1.1027 |
| | Shapiro-Wilk | p-value | 0.1325 | 0.1081 | 0.0113 | 0.0051 | 0.0094 | 0.0703 | 0.8615 | 0.7095 | 0.0116 |
| | | normality | 1 | 1 | 0 | 0 | 0 | 1 | 1 | 1 | 0 |
| | Paired t-test | t-stat | -2.0620 | 2.6609 | 3.8604 | -3.0551 | 3.2356 | 4.0091 | -0.6992 | 1.2362 | 1.7798 |
| | | df | 28 | 28 | 28 | 13 | 13 | 13 | 14 | 14 | 14 |
| | | p-value | 0.0486 | 0.0128 | 0.0006 | 0.0092 | 0.0065 | 0.0015 | 0.4959 | 0.2367 | 0.0968 |
| | Wilcoxon Signed Rank | z-value | -2.2409 | 2.5408 | 2.9759 | -3.0973 | 2.8856 | 3.4871 | -0.7683 | 1.1121 | 1.1005 |
| | | signedrank | 79.5000 | 188.5000 | 292.5000 | 0.0000 | 45.0000 | 78.0000 | 46.0000 | 54.0000 | 70.5000 |
| | | p-value | 0.0250 | 0.0111 | 0.0029 | 0.0020 | 0.0039 | 0.0005 | 0.4442 | 0.2603 | 0.2727 |

Title:
Adaptive Attention-Based Human Machine Interface System for Teleoperation of Industrial Vehicle

Authors:
J.Y. Chew et al.

| | | | All (n=29) | | | Expert (n=14) | | | Beginner (n=15) | | |
|---|---|---|---|---|---|---|---|---|---|---|---|
| | | | UI1 vs UI2 | UI1 vs UI3 | UI2 vs UI3 | UI1 vs UI2 | UI1 vs UI3 | UI2 vs UI3 | UI1 vs UI2 | UI1 vs UI3 | UI2 vs UI3 |
| NASA-TLX (Performance) | Mean of differences | | 16.8103 | -9.5690 | -26.3793 | 20.7143 | -6.4286 | -27.1429 | 13.1667 | -12.5000 | -25.6667 |
| | Stdev of differences | | 21.7319 | 19.0871 | 21.9230 | 20.2491 | 18.6495 | 19.0863 | 23.1159 | 19.6623 | 24.9368 |
| | Median of differences | | 15.0000 | -7.5000 | -22.5000 | 22.5000 | -10.0000 | -26.2500 | 10.0000 | -5.0000 | -20.0000 |
| | Skewness | | 0.0132 | 0.2261 | -0.5594 | -0.0418 | 1.7638 | -0.7210 | 0.1777 | -0.8843 | -0.5610 |
| | Shapiro-Wilk | p-value | 0.4368 | 0.0349 | 0.1188 | 0.5568 | 0.0053 | 0.4082 | 0.3859 | 0.0468 | 0.2237 |
| | | normality | 1 | 0 | 1 | 1 | 0 | 1 | 1 | 0 | 1 |
| | Paired t-test | t-stat | -4.1656 | 2.6998 | 6.4798 | -3.8276 | 1.2898 | 5.3211 | -2.2060 | 2.4622 | 3.9863 |
| | | df | 28 | 28 | 28 | 13 | 13 | 13 | 14 | 14 | 14 |
| | | p-value | 0.0003 | 0.0116 | 0.0000 | 0.0021 | 0.2196 | 0.0001 | 0.0446 | 0.0274 | 0.0014 |
| | Wilcoxon Signed Rank | z-value | -3.4990 | 2.9789 | 4.4887 | -2.7913 | 2.1051 | 3.8419 | -2.2994 | 2.0409 | 3.3345 |
| | | signedrank | 43.5000 | 355.0000 | 400.0000 | 10.0000 | 86.0000 | 105.0000 | 13.0000 | 96.0000 | 100.5000 |
| | | p-value | 0.0005 | 0.0029 | 0.0000 | 0.0050 | 0.0345 | 0.0001 | 0.0200 | 0.0388 | 0.0010 |
| NASA-TLX (Effort) | Mean of differences | | 10.1724 | -7.5862 | -17.7586 | 12.6786 | -7.5000 | -20.1786 | 7.8333 | -7.6667 | -15.5000 |
| | Stdev of differences | | 17.0206 | 14.0865 | 23.1681 | 15.7951 | 13.9711 | 23.8880 | 18.3193 | 14.6832 | 23.0721 |
| | Median of differences | | 7.5000 | -5.0000 | -17.5000 | 10.0000 | -6.2500 | -16.2500 | 7.5000 | -5.0000 | -20.0000 |
| | Skewness | | -0.3081 | -0.9210 | 0.1599 | 0.3237 | -0.5862 | -0.2128 | -0.6068 | -1.2816 | 0.5874 |
| | Shapiro-Wilk | p-value | 0.5428 | 0.0139 | 0.7192 | 0.9605 | 0.4090 | 0.8841 | 0.3440 | 0.0264 | 0.6246 |
| | | normality | 1 | 0 | 1 | 1 | 1 | 1 | 1 | 0 | 1 |
| | Paired t-test | t-stat | -3.2185 | 2.9001 | 4.1278 | -3.0034 | 2.0086 | 3.1606 | -1.6561 | 2.0222 | 2.6019 |
| | | df | 28 | 28 | 28 | 13 | 13 | 13 | 14 | 14 | 14 |
| | | p-value | 0.0032 | 0.0072 | 0.0003 | 0.0102 | 0.0658 | 0.0075 | 0.1199 | 0.0627 | 0.0209 |
| | Wilcoxon Signed Rank | z-value | -3.0527 | 2.4085 | 3.4496 | -2.6017 | 1.5594 | 2.7107 | -1.8958 | 1.7595 | 2.3848 |
| | | signedrank | 55.5000 | 308.5000 | 332.5000 | 7.5000 | 78.0000 | 93.5000 | 22.0000 | 81.0000 | 79.0000 |
| | | p-value | 0.0023 | 0.0160 | 0.0006 | 0.0107 | 0.1152 | 0.0073 | 0.0564 | 0.0757 | 0.0164 |
| NASA-TLX (Frustration) | Mean of differences | | 16.2069 | -8.0172 | -24.2241 | 19.4643 | -10.8929 | -30.3571 | 13.1667 | -5.3333 | -18.5000 |
| | Stdev of differences | | 21.0825 | 11.3661 | 23.6521 | 19.2698 | 12.6570 | 21.2326 | 22.8830 | 9.6763 | 25.0500 |
| | Median of differences | | 12.5000 | -5.0000 | -22.5000 | 12.5000 | -8.7500 | -27.5000 | 10.0000 | -2.5000 | -12.5000 |
| | Skewness | | 0.2180 | -1.0142 | 0.1969 | 1.1373 | -0.4449 | -0.1972 | -0.1224 | -1.9389 | 0.2430 |
| | Shapiro-Wilk | p-value | 0.3605 | 0.0226 | 0.9096 | 0.0312 | 0.9394 | 0.9876 | 0.9862 | 0.0014 | 0.3612 |
| | | normality | 1 | 0 | 1 | 0 | 1 | 1 | 1 | 0 | 1 |
| | Paired t-test | t-stat | -4.1398 | 3.7985 | 5.5154 | -3.7794 | 3.2201 | 5.3496 | -2.2285 | 2.1347 | 2.8603 |
| | | df | 28 | 28 | 28 | 13 | 13 | 13 | 14 | 14 | 14 |
| | | p-value | 0.0003 | 0.0007 | 0.0000 | 0.0023 | 0.0067 | 0.0001 | 0.0428 | 0.0509 | 0.0126 |
| | Wilcoxon Signed Rank | z-value | -3.6558 | 3.3768 | 3.9644 | -3.6683 | 2.7107 | 3.5633 | -2.0348 | 2.1416 | 2.4722 |
| | | signedrank | 37.0000 | 287.5000 | 377.0000 | 0.0000 | 93.5000 | 103.0000 | 20.0000 | 57.0000 | 91.0000 |
| | | p-value | 0.0003 | 0.0007 | 0.0001 | 0.0002 | 0.0073 | 0.0004 | 0.0403 | 0.0293 | 0.0132 |
